# Tricyclic Antidepressants Promote Ceramide Accumulation to Regulate Collagen Production in Human Hepatic Stellate Cells

**DOI:** 10.1038/srep44867

**Published:** 2017-03-21

**Authors:** Jennifer Y. Chen, Benjamin Newcomb, Chan Zhou, Joshua V. Pondick, Sarani Ghoshal, Samuel R. York, Daniel L. Motola, Nicolas Coant, Jae Kyo Yi, Cungui Mao, Kenneth K. Tanabe, Irina Bronova, Evgeny V. Berdyshev, Bryan C. Fuchs, Yusuf Hannun, Raymond T. Chung, Alan C. Mullen

**Affiliations:** 1 Gastrointestinal Unit, Massachusetts General Hospital, Harvard Medical School, Boston, MA USA; 2 Health Science Center, Stony Brook University, Stony Brook, NY USA; 3 Division of Surgical Oncology, Massachusetts General Hospital Cancer Center, Harvard Medical School, Boston, MA USA; 4 National Jewish Health, Denver, CO USA; 5 Harvard Stem Cell Institute, Cambridge, MA 02138 USA

## Abstract

Activation of hepatic stellate cells (HSCs) in response to injury is a key step in hepatic fibrosis, and is characterized by trans-differentiation of quiescent HSCs to HSC myofibroblasts, which secrete extracellular matrix proteins responsible for the fibrotic scar. There are currently no therapies to directly inhibit hepatic fibrosis. We developed a small molecule screen to identify compounds that inactivate human HSC myofibroblasts through the quantification of lipid droplets. We screened 1600 compounds and identified 21 small molecules that induce HSC inactivation. Four hits were tricyclic antidepressants (TCAs), and they repressed expression of pro-fibrotic factors Alpha-Actin-2 (*ACTA2*) and Alpha-1 Type I Collagen (*COL1A1)* in HSCs. RNA sequencing implicated the sphingolipid pathway as a target of the TCAs. Indeed, TCA treatment of HSCs promoted accumulation of ceramide through inhibition of acid ceramidase (aCDase). Depletion of aCDase also promoted accumulation of ceramide and was associated with reduced *COL1A1* expression. Treatment with B13, an inhibitor of aCDase, reproduced the antifibrotic phenotype as did the addition of exogenous ceramide. Our results show that detection of lipid droplets provides a robust readout to screen for regulators of hepatic fibrosis and have identified a novel antifibrotic role for ceramide.

Fibrosis is the common endpoint for all etiologies of chronic liver disease. It is the progression of fibrosis that leads to end stage liver disease, which affects approximately 600,000 Americans and accounts for 30,000 deaths each year^1^,^2^. Despite our understanding that progressive fibrosis is the major cause of liver failure^3^,^4^, there remains no effective treatment targeted at preventing this process.

Hepatic stellate cells (HSCs) are the primary cell type responsible for liver fibrosis^5^,^6^. In their quiescent state, HSCs serve as a reservoir for vitamin A, which is stored in lipid droplets^7^. HSCs are activated in normal wound repair; however, upon repeated activation in response to chronic injury, HSCs trans-differentiate into HSC myofibroblasts, leading to loss of lipid droplets and induction of contractile filaments such as Alpha-Actin-2 (α-SMA, encoded by *ACTA2*) and extracellular matrix (ECM) proteins such as Alpha-1 Type I Collagen (COL1A1)^3^,^8^. Increased expression of α-SMA confers increased contractile potential, and type 1 collagen is the best-studied component of the hepatic scar that is regulated by HSC myofibroblasts^8^. The deposition and cross-linking of ECM by HSC myofibroblasts lead to hepatic fibrosis and subsequent liver failure^5^,^9^,^10^,^11^.

Studies using cell fate tracking have demonstrated that 40–50% of HSC myofibroblasts revert to an inactive phenotype *in vivo* during fibrosis regression^12^,^13^. HSC myofibroblasts can also be induced to revert to an inactive phenotype in cell culture^14^,^15^. These inactive HSCs are characterized by reduced expression of *ACTA2* and *COL1A1*^14^,^15^, induction of adipogenic genes including peroxisome proliferator-activator receptor gamma (*PPAR-γ*)^16^,^17^, and acquisition of lipid droplets^15^, phenotypes that are shared with quiescent HSCs^5^,^18^.

To identify novel targets in hepatic fibrosis, we developed a small molecule screen to detect compounds that induce reversion of primary human HSC myofibroblasts to an inactive phenotype, as defined by accumulation of lipid droplets. This approach revealed the antifibrotic effects of tricyclic antidepressants (TCAs) and identified accumulation of ceramide as a key event mediating this activity. TCAs promote accumulation of ceramide through inhibition of acid ceramidase (aCDase), an enzyme in the sphingolipid salvage pathway, and this study shows that inhibition of aCDase is a promising therapeutic strategy to reverse liver fibrosis.

## Results

### A small molecule screen identifies TCAs as inhibitors of hepatic fibrosis

**Figure 1:**
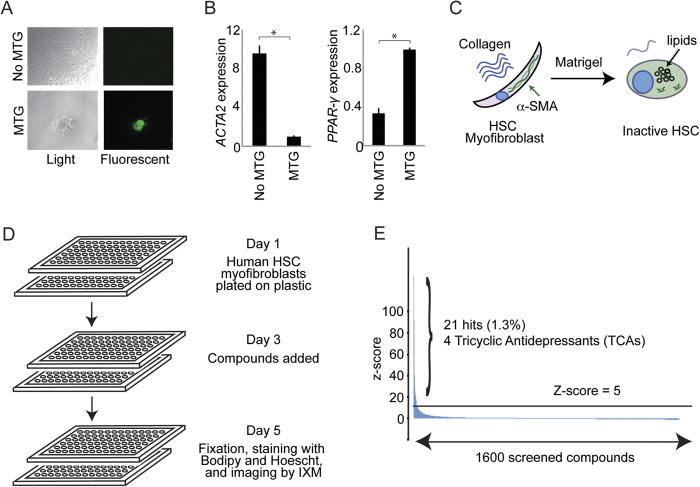
Design and performance of a small molecule screen to identify compounds that inhibit hepatic fibrosis. (**A**) Culture in Matrigel (MTG) leads to accumulation of lipid droplets characteristic of inactive HSCs. Light microscopy images (left) are shown for HSC myofibroblasts cultured without MTG (top) and with MTG (bottom) for 3 days. Staining with Bodipy 493/503 for neutral fat (right) shows accumulation of lipid droplets (green) in HSCs cultured in MTG (lower right), and not in HSCs cultured without MTG (upper right). Images are shown at 10x magnification. (**B**) Quantitative real time (qRT)-PCR was performed to measure *ACTA2* (left) and *PPAR-γ* (right) mRNA levels in HSC myofibroblasts with and without MTG for 3 days. Samples were normalized using *GAPDH.* *p < 0.05. (**C**) Culture of human HSC myofibroblasts in MTG leads to accumulation of lipid droplets and reduced expression of α-SMA (encoded by *ACTA2*). (**D**) Schematic illustrating the screen designed to identify compounds that revert HSC myofibroblasts to inactive HSCs. (**E**) 1600 known bioactive compounds were screened, and the Median Absolute Deviation (MAD)-based Z score (y-axis) was plotted for each compound (x-axis). The horizontal black line indicates a MAD-based Z score of 5.

We first isolated quiescent human HSCs from excess liver tissue and differentiated the cells into HSC myofibroblasts by culture on plastic^5^,^19^,^20^. Culturing these HSC myofibroblasts in Matrigel^15^ led to accumulation of lipid droplets (Fig. 1A), reduced *ACTA2* expression, and increased *PPAR-γ* expression (Fig. 1B), which are characteristic of inactive HSCs (Fig. 1C). We then developed a high throughput screen to quantify HSC inactivation by measuring lipid droplet formation. Lipid droplets were quantified using Bodipy, a fluorescent, cell-permeable lipid dye that has been utilized to identify quiescent HSCs^21^. HSC myofibroblasts were plated on day 1, treated with compounds on day 3, and analyzed for lipid droplet formation on day 5 by the Image Xpress Micro (IXM) (Fig. 1D). Cells grown in Matrigel served as positive controls while cells treated with 0.03% v/v dimethyl sulfoxide (DMSO) alone were used as negative controls. Compounds with a Median Absolute Deviation (MAD)-based Z score greater than 5 in two replicates were considered positive hits (Fig. 1E), and those associated with more than 60% cell death were excluded. Twenty-one hits were identified from a library of 1600 known bioactive compounds (Table 1). Strikingly, four hits were tricyclic antidepressants (TCAs). The screen also identified chloroquine, which has been shown to reduce hepatic fibrosis *in vivo*^22^.

### TCAs inactivate HSCs and suppress ECM production

**Figure 2:**
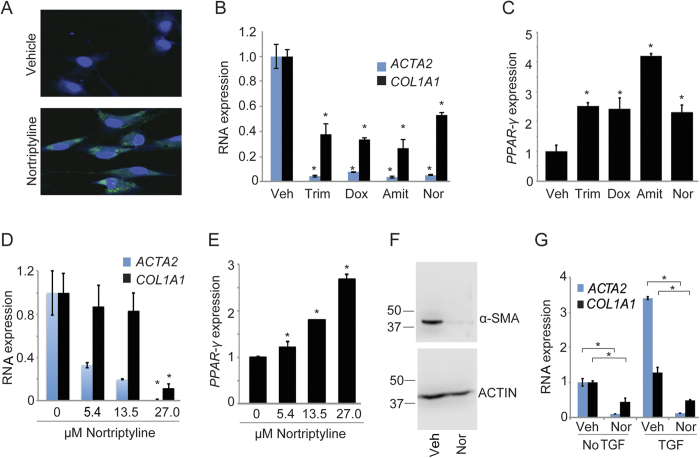
TCAs induce an inactive HSC phenotype. (**A**) Lipid accumulation was assessed in HSCs treated with ethanol vehicle or nortriptyline (27 μM, unless otherwise specified) for 48 hours. Cells were stained with Bodipy (green) and Hoescht (blue). (**B** and **C**) qRT-PCR was performed for the indicated genes. HSCs were treated with DMSO (veh), trimipramine (Trim, 24 uM), doxepin (Dox, 25 μM), amitriptyline (Amit, 25 μM), and nortriptyline (Nor) for 48 hours. Samples were normalized using *GAPDH*. *p < 0.05. (**D** and **E**) qRT-PCR was performed to quantify expression of the indicated genes following nortriptyline treatment at the indicated concentrations for 48 hours. (**F**) α-SMA was measured by Western blot from HSCs treated with ethanol vehicle or nortriptyline for 48 hours. ACTIN serves as a loading control. Cropped gel images are shown. Molecular weight markers in kilodaltons (kD) are shown on the left of each blot. (**G**) *ACTA2* and *COL1A1* levels were quantified in HSCs that were serum-starved for 48 hours and then treated with ethanol vehicle or nortriptyline for 16** **hours in the presence (right) or absence (left) of TGF-β (2.5 ng/ml).

The effects of TCAs were investigated further in a second human HSC myofibroblast line isolated from human liver tissue. Treatment with nortriptyline, the TCA with the highest MAD-based Z score, was associated with accumulation of lipid droplets visualized by Bodipy staining compared to HSCs receiving vehicle (Fig. 2A). Treatment of HSC myofibroblasts with four different TCAs inhibited *ACTA2* and *COL1A1* (Fig. 2B) while inducing *PPAR-γ* expression (Fig. 2C). Amitriptyline was associated with a lower MAD-based Z score (2.5), but was selected for verification given its structural similarity to nortriptyline. In addition, the response to nortriptyline was dose-dependent (Fig. 2D,E), and reduced expression of *ACTA2* mRNA by nortriptyline was associated with a decrease in α-SMA protein (Fig. 2F). Nortriptyline also suppressed expression of *ACTA2* and *COL1A1* following treatment with transforming growth factor-beta (TGF-β) (Fig. 2G), a potent stimulus of fibrosis^23^.

**Figure 3:**
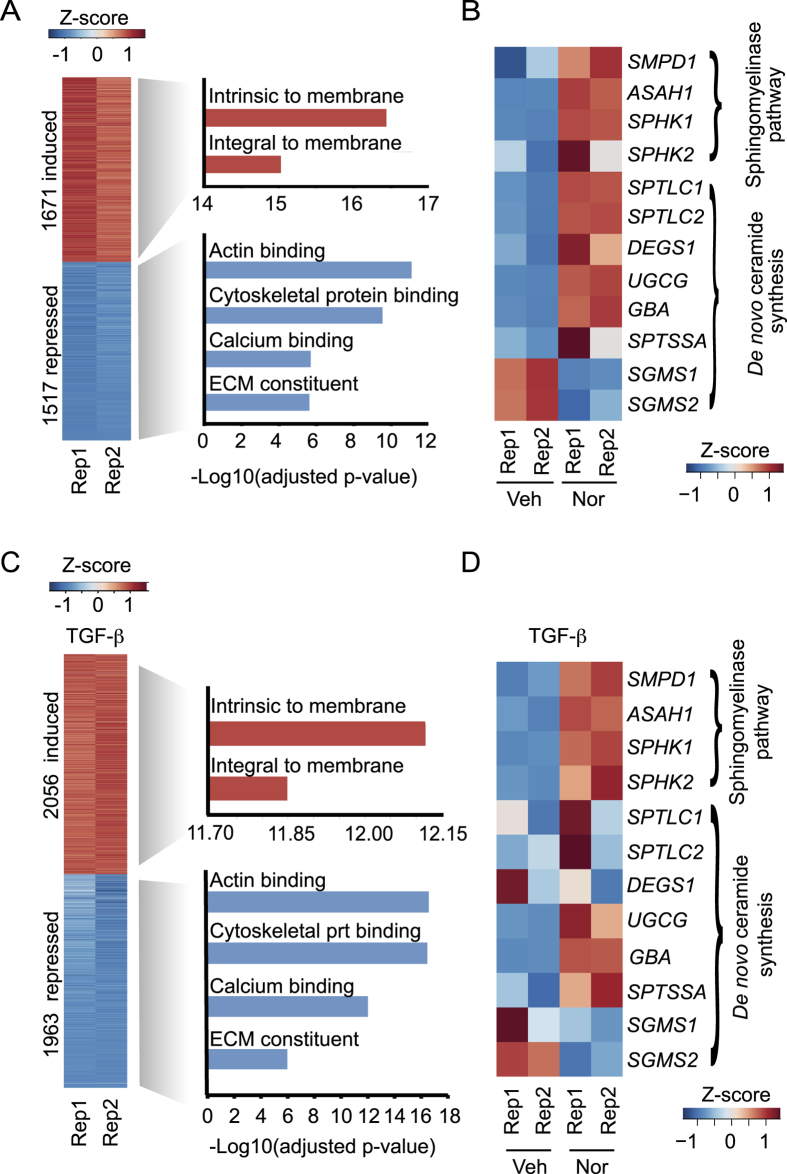
TCA treatment inhibits extracellular matrix constituents and regulates genes in the sphingomyelinase pathway. (**A**) RNA-seq was performed on HSCs treated with ethanol vehicle or nortriptyline for 48 hours. Protein-coding genes induced (red) and repressed (blue) compared to vehicle are shown (at least 1.5 fold change in expression, false discovery rate < 0.0001). The relative expression (Z-score) of two replicates (Rep 1 and Rep 2) is shown. The most significant categories identified by GO analysis are shown for genes that were induced (red) or repressed (blue). (**B**) Expression of genes involved in the sphingomyelinase and *de novo* ceramide pathways following treatment with vehicle or nortriptyline. The relative expression (Z-score) is shown for two replicates (Rep 1 and Rep 2). (**C**) RNA-seq was performed on HSCs that were serum starved for 48 hours prior to treatment with TGF-β (2.5 ng/ml) and either ethanol vehicle or nortriptyline (27 μM) for 16 hours. Protein-coding genes that were induced (red) and repressed (blue) after nortriptyline treatment compared to vehicle are shown (at least 1.5 fold change in expression, false discovery rate < 0.0001). Relative expression (Z-score) for two replicates (Rep 1 and Rep 2) is shown. The most significant categories identified by GO analysis are shown for genes that were induced (red) or repressed (blue). (**D**) Expression of genes involved in the sphingolipid pathway following treatment with vehicle or nortriptyline in the presence of TGF-β. Relative expression (Z-score) is shown for two replicates (Rep 1 and Rep 2).

To understand how TCAs induce inactivation of HSCs, we performed RNA sequencing (seq) on HSC myofibroblasts treated with nortriptyline. We found that 1671 protein-coding genes were induced by nortriptyline treatment, while 1517 were repressed (Fig. 3A and Data file S1). Gene ontology analysis revealed that the genes repressed by nortriptyline were most enriched in the functional categories of actin-binding, cytoskeletal proteins and ECM constituents (Fig. 3A). These findings show that nortriptyline broadly inhibits expression of genes involved in fibrosis in addition to *COL1A1*. The genes induced by nortriptyline were most enriched in the category intrinsic to membrane (Fig. 3A), which included genes involved in sphingolipid metabolism. We found that nortriptyline regulated expression of many genes involved in the sphingomyelinase pathway and in *de novo* ceramide synthesis (Fig. 3B), including acid sphingomyelinase (encoded by *SMPD1*)^24^ and acid ceramidase (encoded by *ASAH1*)^25^. Similar results were observed when HSCs stimulated with TGF-β were treated with nortriptyline (Fig. 3C,D and Data file S1). In addition, 63 long noncoding (lnc) RNAs were induced and 130 lncRNAs were repressed by nortriptyline (Supplemental Fig. 1A and Data file S1). Co-expression network analysis^26^ identified protein-coding and lncRNA genes repressed by nortriptyline that are contained in networks with ECM genes (Supplemental Fig. 1B and Data file S1), suggesting that TCAs suppress expression of both protein-coding and lncRNA genes linked to fibrosis.

### TCAs inactivate HSCs by aCDase inhibition

**Figure 4:**
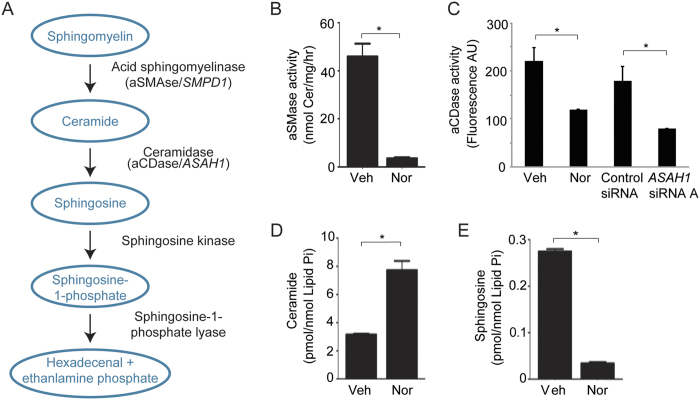
TCAs inhibit acid ceramidase in human HSCs, leading to accumulation of ceramide. (**A**) Diagram of the sphingomyelinase pathway. (**B**) aSMase enzyme assay results of HSCs treated with ethanol vehicle (Veh) or nortriptyline (Nor, 27 μM) for 48 hours. *p < 0.05. (**C**) aCDase enzyme assay results of HSCs treated with ethanol vehicle (Veh) or nortriptyline (Nor, 27 μM) for 48 hours and HSCs transfected with nontargeting siRNA (control) or siRNA targeting aCDase mRNA (*ASAH1*). *p < 0.05. (**D** and **E**) Ceramide and sphingosine accumulation was measured in HSCs treated with vehicle or nortriptyline. *p < 0.05.

RNA-seq results identified the sphingomyelinase and ceramide synthesis pathways as targets of TCAs, and TCAs have been shown to inhibit both acid sphingomyelinase (aSMase) and acid ceramidase (aCDase)^27^,^28^,^29^,^30^,^31^. aSMase is a soluble hydrolase that cleaves the phosphodiester bond of sphingomyelin to form the bioactive lipid ceramide. Deacylation of ceramide by aCDase produces sphingosine, another bioactive sphingolipid; in turn, phosphorylation of sphingosine by sphingosine kinase produces the pro-mitogenic sphingosine-1-phosphate (Fig. 4A)^32^. Ceramide and sphingosine influence various processes including apoptosis, autophagy, senescence, differentiation, protein trafficking, migration, and proliferation^33^,^34^,^35^,^36^. Given studies have shown that TCAs inhibit both aSMase and aCDase in other cell types^27^,^28^,^29^,^30^,^31^, we next investigated how TCAs affect the sphingomyelinase pathway and how individual components of this pathway regulate the fibrotic activity of human HSC myofibroblasts.

We found that the enzymatic activities of aSMase and aCDase were significantly reduced in HSCs treated with nortriptyline compared to vehicle (Fig. 4B,C). In addition, the reduction in aCDase activity by nortriptyline was similar to that observed in HSCs depleted of aCDase (*ASAH1*) (Fig. 4C and Supplemental Fig. 2). To determine the functional effects of inhibition of these enzymes, we next performed sphingolipid analysis of nortriptyline-treated human HSCs. The results showed an accumulation of total ceramide and a reduction in sphingosine (Fig. 4D,E and Data file S2), which suggested that aCDase is a primary target of nortriptyline in HSCs.

**Figure 5:**
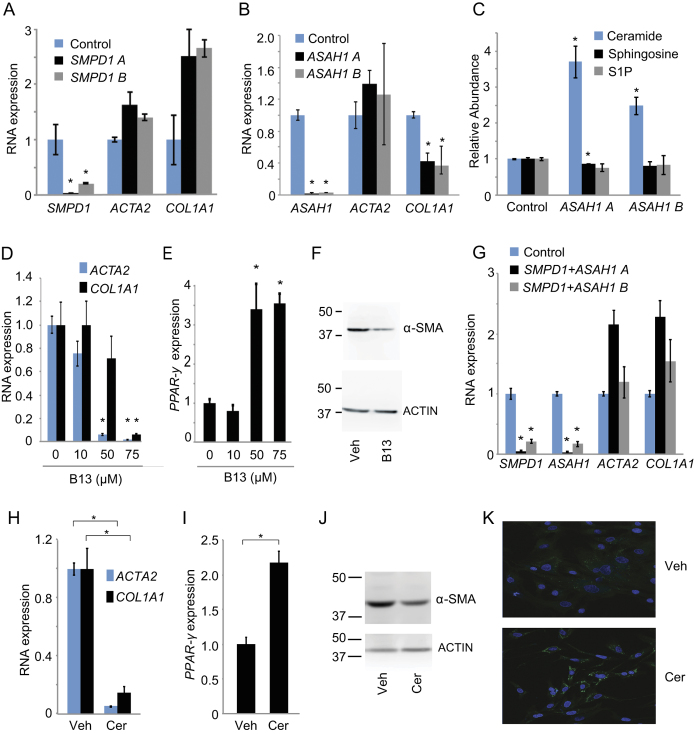
Ceramide regulates collagen expression. (**A** and **B**) qRT-PCR was performed to quantify expression of the indicated genes after transfection with nontargeting siRNA (control) and siRNAs targeting aSMase mRNA (*SMPD1*) and aCDase mRNA (*ASAH1*). Two siRNAs (A and B) were used for each mRNA. Samples were normalized using *GAPDH*. *p < 0.05. (**C**) Ceramide, sphingosine, and sphingosine 1-phosphate (S1P) were measured in HSCs transfected with a nontargeting siRNA (control) or siRNAs targeting aCDase mRNA (*ASAH1*). Metabolite levels are shown relative to siRNA controls, which were set to 1. *p < 0.05. (**D** and **E**) qRT-PCR was performed to quantify expression of the indicated genes after treatment with ethanol vehicle and B13 at the indicated concentrations. (**F**) α-SMA expression was quantified by Western blot from HSCs treated with ethanol vehicle (Veh) or B13 (75 μM). ACTIN is shown as a loading control. Molecular weight markers in kilodaltons (kD) are shown on the left of each blot. Cropped gel images are shown. (**G**) qRT-PCR was performed to quantify expression of the indicated genes after transfection with nontargeting siRNA (control) and siRNAs targeting aSMase mRNA (*SMPD1*) and aCDase mRNA (*ASAH1*) in combination. Two siRNAs (A and B) were used for each mRNA. Samples were normalized using *GAPDH*. *p < 0.05. (**H** and **I**) qRT-PCR was performed to quantify expression of the indicated genes after treatment with ethanol vehicle and ceramide-C6 (25 μM). Samples were normalized using *GAPDH*. *p < 0.05. (**J**) α-SMA expression was quantified by Western blot from HSCs treated with ethanol vehicle (Veh) or ceramide-C6 (25 μM). ACTIN is shown as a loading control. Molecular weight markers in kilodaltons (kD) are shown on the left of each blot. Cropped gel images are shown. (**K**) Lipid accumulation was assessed in HSCs treated with ethanol vehicle or ceramide-C6 (25 μM) for 48 hours. Cells were stained with Bodipy (green) and Hoescht (blue).

Since both aSMase and aCDase are targets of TCAs, we conducted experiments to define the roles of each enzyme in human HSC myofibroblasts. We transfected cells with siRNAs targeting *SMPD1* (aSMase), *ASAH1* (aCDase), and the transcripts encoding sphingosine kinase 1 (*SPHK1*) and 2 (*SPHK2*). Depletion of *SMPD1* did not lead to a reduction in *ACTA2* or *COL1A1* expression (Fig. 5A). However, we observed a reduction in *COL1A1* expression with depletion of *ASAH1* (Fig. 5B) while depletion of *SPHK1* or *SPHK2* did not reduce *ACTA2* or *COL1A1* levels (Supplemental Fig. 3A,B). Similarly, depletion of both *SPHK1* and *SPHK2* together did not reduce *ACTA2* or *COL1A1* levels (Supplemental Fig. 3C). Moreover, depletion of *ASAH1* by two independent siRNAs led to a significant increase in total ceramide levels (Fig. 5C and Data file S3). The accumulation of total ceramide in HSCs depleted of *ASAH1* was similar to the level observed in HSCs depleted of *ASAH1* and treated with nortriptyline (Supplemental Fig. 3D). Taken together, these results suggest that TCAs exert their antifibrotic effect in human HSCs through inhibition of aCDase and that aCDase inhibition is sufficient to explain the increase in ceramide observed with TCA treatment.

To provide further evidence that inhibition of aCDase was sufficient to revert HSCs to an inactive phenotype, we treated HSC myofibroblasts with B13^37^, a small molecule inhibitor of aCDase. Treatment with B13 was associated with a dose-dependent reduction in *ACTA2* and *COL1A1* expression and activation of *PPAR-γ* (Fig. 5D,E). Treatment with B13 was also associated with decreased α-SMA protein levels (Fig. 5F). aCDase inhibitors can induce apoptosis in cancer cells^38^, but HSCs treated with 50 μM and 75 μM of B13 demonstrated only 1.2% and 2.6% apoptosis, respectively (Supplemental Fig. 3E), suggesting that B13 treatment does not induce the inactive HSC phenotype through apoptosis.

TCAs are described to inhibit both aSMase and aCDase, and we next asked how simultaneous depletion of both enzymes affected collagen expression. We observed that depletion of *SMPD1* and *ASAH1* together prevented the repression of *COL1A1* (Fig. 5G), which occurred with depletion of *ASAH1* alone (Fig. 5B). A two-way ANOVA was calculated, and there was no significant interaction between depletion of *SMPD1, ASAH1*, and depletion of both *SMPD1* and *ASAH1* on *COL1A1* expression (p = 0.78). This finding suggested that accumulation of ceramide may be responsible for the phenotype observed when *ASAH1* is depleted alone. To test if accumulation of ceramide promotes inactivation, we treated HSCs with exogenous ceramide-C6 and found that ceramide treatment led to a reduction in *ACTA2* and *COL1A1* expression (Fig. 5H). This effect was dose-dependent (Supplemental Fig. 3F), and treatment with ceramide also led to activation of *PPAR-γ* (Fig. 5I), reduction in α-SMA protein levels (Fig. 5J), and lipid accumulation (Fig. 5K). Indeed, the overall pattern of genes induced and repressed by ceramide (Supplemental Fig. 3G and Data File S1) mirrored the pattern observed with nortriptyline treatment (Fig. 3A). Ceramide treatment resulted in minimal evidence of apoptosis or necrosis (Supplemental Fig. 3H), and when viable cells were sorted (Annexin V and Propidium Iodide negative), the effects of ceramide on *ACTA2, COL1A1,* and *PPAR-γ* expression persisted (Supplemental Fig. 3I,J), further suggesting that ceramide does not promote HSC inactivation through apoptosis or necrosis. Taken together, these results suggest that ceramide accumulation through TCA treatment, aCDase inhibition, or addition of exogenous ceramide, leads to HSC inactivation.

## Discussion

Despite significant advances in our understanding of hepatic fibrogenesis, including the key role of the HSC myofibroblast, there remains no effective therapy targeting the common endpoint of fibrosis. Therefore, we performed a screen to identify small molecules that inactivate HSCs with the goal of identifying pathways that regulate the fibrotic response. Our finding that TCAs inactivate human HSC myofibroblasts along with studies showing that TCAs can inhibit hepatic fibrosis in mouse models^39^,^40^ suggest that TCAs may regulate fibrosis by inactivating HSC myofibroblasts and that downstream targets of TCAs could form the basis of future therapies to treat fibrosis.

TCAs have variable affinity for antagonizing 5-hydroxytryptamine, muscarinic acetylcholine, adrenergic, histamine H1 and H2 receptors, and the serotonin and norepinephrine transporters^41^,^42^ in addition to regulating the sphingomyelinase pathway^27^,^28^,^29^. We focused on the sphingomyelinase pathway due to the ability of TCAs to affect expression of enzymes in this pathway and previous studies suggesting that this pathway may play a role in liver fibrosis.

Prior studies have shown that TCAs inhibit the enzymatic activity of aSMase and aCDase. The TCA desipramine decreases aSMase activity by interfering with the binding of aSMase to the lipid bilayers of the lysosome, which displaces the enzyme from its membrane-bound substrate and results in intracellular degradation of the mature enzyme^30^,^31^. Additional inhibitors of aSMase were identified using a structure-property-activity relation model, and these inhibitors included the TCAs nortriptyline and doxepin^43^, which were both identified in our screen. TCAs were later recognized to inhibit aCDase in a dose- and time-dependent manner^28^,^29^. Treatment with despiramine resulted in ceramide accumulation and a reduction in sphingosine^29^, which is consistent with our findings in human HSCs.

Our results confirm that TCAs affect both aSMase and aCDase in human HSC myofibroblasts and extend the findings to show that while both enzymes are inhibited, it is the inhibition of aCDase and the resulting accumulation of ceramide that promotes inactivation of HSCs. Increased aCDase activity has also been observed with induction of liver fibrosis *in vivo*^44^, suggesting that increased aCDase activity could serve as a marker of fibrosis. While no studies have investigated the effect of inhibition of aCDase in models of hepatic fibrosis, aCDase inhibitors have been well-tolerated in mouse tumor models^37^,^38^, and future studies to define the antifibrotic effect of aCDase inhibition *in vivo* will be of great interest.

Depletion of the other enzymes in the sphingomyelinase pathway (aSMase, SPHK1, and SPHK2) did not lead to a reduction in *COL1A1* mRNA, and review of the literature in large part corroborates these findings. For example, Moles *et al*. observed that depletion of aSMase did not lead to a reduction in *COL1A1* mRNA, which is consistent with our results^45^. The authors did observe a reduction in *ACTA2* expression, which was not observed in our study; however, they used HSCs isolated from mice rather than human HSCs, which may account for this difference. Similarly, Xiu *et al*. tested siRNAs targeting *SPHK1* in human HSCs^46^. Consistent with our findings, depletion of *SPHK1* did not result in a reduction in *COL1A1* mRNA levels compared to a control siRNA; moreover, treatment with N,N-dimethylsphingosine (DMS), an inhibitor of sphingosine kinase, did not lead to *COL1A1* mRNA reduction. The authors did observe a reduction in *COL1A1* mRNA in the presence of siRNA targeting *SPHK1* or DMS treatment in response to TGF-β1, and they concluded that intracellular S1P may play an important role in the TGF-β1-induced expression of *COL1A1*. Similar findings were observed by Gorshkova *et al*. using primary human lung fibroblasts^47^. Our study did not investigate the additional effects of TGF-β on regulation of HSC activation by the enzymes of the sphingomyelinase pathway.

The role of ceramide in hepatic fibrosis has not been described previously; however, there are data on the potential antifibrotic role of S1P, a sphingolipid metabolite downstream of ceramide (Fig. 4A), which deserve mention in light of our findings. The addition of S1P to LX2 cells, an immortalized HSC line^48^, led to increased *ACTA2* and *COL1A1* expression through S1P Receptor (R) 1 and S1PR3 but not S1PR2^49^. In the carbon tetrachloride-induced model of hepatic fibrosis, S1PR2-deficient mice experienced decreased fibrosis^50^. S1PR3 expression was elevated in cholestasis-induced hepatic fibrosis, and S1PR3 inhibition led to reduced fibrosis in this model^51^. Variations in circulating and liver S1P levels and S1P receptor expression patterns have been observed in humans with chronic liver disease. In one study, mRNA levels of sphingosine kinase 1 and S1PR2 correlated with fibrosis stages in human liver fibrosis^52^. However, in another study, S1PR1 and S1PR3 were strongly induced, whereas expression of S1PR2 was decreased in human liver fibrosis^53^. Conflicting data exist regarding the correlation between plasma S1P levels and fibrosis. Plasma S1P levels were found to be reduced in patients with chronic hepatitis C infection in association with fibrosis^54^. However, in a study of healthy volunteers, patients with non-alcoholic fatty liver disease (NAFLD), and patients with chronic hepatitis C infection, variation in S1P was not observed, but there were elevations in sphingosine levels in those with chronic liver disease compared to healthy individuals^55^. Similarly, in a study among patients with chronic viral hepatitis B and C, there was no correlation between liver fibrosis stage and serum S1P^56^. In this study, decreased sphingosine levels were associated independently with fibrosis progression. In summary, the association between sphingolipid levels and fibrosis progression among patients with chronic liver disease remains unclear.

Our study focused on elucidating how TCAs regulate the sphingomyelinase pathway to mediate HSC activation, and through detailed sphingolipid analysis and the use of siRNAs and small molecule inhibitors, we have identified a potential antifibrotic role of ceramide. The mechanism by which ceramide leads to HSC inactivation requires further investigation, and it will be important to determine whether the effects of ceramide are mediated through downregulation of S1P signaling. Sphingolipid analysis following depletion of aCDase shows a trend towards a slight reduction in sphingosine and S1P, but these are not statistically significant in replicates (Fig. 5C). Our results also show that inhibition of SPHK1 and SPHK2 (independently or in combination) (Supplemental Fig. 3A,B and C) do not reduce collagen levels in human HSCs. It is possible that ceramide accumulation may regulate S1P receptors to control S1P signaling, and further studies are also necessary to determine how ceramide accumulation in HSCs alters sphingolipid metabolites in total liver and serum.

We found that nortriptyline induced expression of the genes encoding aSMase and aCDase (Fig. 3B), and increased expression of the aSMase precursor was also observed following treatment with desipramine^27^. We speculate that the transcriptional response may be activated to compensate for the decreased enzymatic activity of aSMase and aCDase in the presence of TCAs. Nortriptyline also induced expression of other enzymes that facilitate production of ceramide, including serine palmitoyltransferase (SPT), the rate-limiting enzyme in the *de novo* synthesis of sphingolipids^57^,^58^; dihydroceramide desaturase (DEGS), which converts dihydroceramide to ceramide^59^; and glucosyl ceramidase (GBA), which converts glucosylceramide to ceramide^60^. Similarly, nortriptyline suppressed expression of sphingomyelin synthase (SGMS), which promotes the conversion of ceramide to sphingomyelin^61^. This suggests that several pathways promoting ceramide production may be enhanced by nortriptyline treatment. However, nortriptyline also induced enzymes involved in sphingolipid metabolism that decrease production of ceramide, including UDP-glucose ceramide glucosyltransferase (UGCG), which converts ceramide to glucosylceramide^62^, and sphingosine kinase 1 (SPHK1), which promotes conversion of ceramide to sphingosine^63^. The mRNA expression of each enzyme may not correlate with enzyme activity: for example, our results show that nortriptyline treatment led to increased expression of the mRNA encoding aCDase but suppressed enzymatic activity in human HSCs. TCAs have been described to inhibit aSMase and aCDase as detailed above^28^,^29^,^30^,^31^ but their effects on other sphingolipid enzymes have not been described. Additional studies are warranted to elucidate the interaction between mRNA expression, protein level, and activity of enzymes involved in sphingolipid metabolism that are regulated by TCAs.

In summary, we have demonstrated that use of Bodipy fluorescence to detect formation of lipid droplets provides a robust readout to screen for compounds that revert HSC myofibroblasts to an inactive HSC phenotype. Using our novel screening methodology, we observed a class effect of TCAs and showed that TCAs not only promote lipid accumulation but also inhibit expression of components of the ECM that regulate fibrosis. Furthermore, we identified a novel antifibrotic role for inhibition of aCDase, which is mediated through accumulation of ceramide. Our findings suggest that pharmacologic inhibition of aCDase represents a promising new therapeutic approach to the treatment of liver fibrosis.

## Materials and Methods

### Ethics Approval

The study protocol to isolate hepatic stellate cells from excess surgical tissue and from human nonparenchymal liver cells (NPCs) purchased from Triangle Research Laboratories (TRL) was reviewed and approved by the Massachusetts General Hospital Institutional Review Board (IRB), and the methods were carried out in accordance with the approved guidelines. A waiver of informed consent was approved by the IRB for use of excess tissue.

### Cell Culture

Adult human HSCs were isolated from human liver tissue samples or from human NPCs obtained from TRL. Fresh human liver samples were placed in cold Dulbelcco’s Phosphate Buffered Saline Solution (dPBS). The tissue was perfused first with dPBS and then placed in a 37 °C digestion solution of dBPS with 0.375 mg/mL of Trypsin Inhibitor (Sigma), 0.536 mg/mL of Collagenase Type 2 (Worthington), and 1.0 mg/mL of Pronase E (OmniPur). Cells were scraped and then filtered using a 100 μm cell filter (Falcon). NPCs from the filtrate or from TRL were centrifuged at 50 *g* for 5 minutes at 4 °C. Cells from the supernatant were pelleted at 860 *g* for 10 min at 4 °C and resuspended in a 60% w/v Optiprep solution (Sigma) diluted to 15% w/v with Hanks Balance Salt Solution without calcium or magnesium and 2.0 mg/mL of DNaseI (Roche). Layers of 11.5% and 8.5% Optiprep solution were added to the 15% layer before centrifugation at 1400 *g* for 17 min with no brake at 4 °C. HSCs were removed from between the 11.5% and 8.5% layers and pelleted at 860 *g* for 5 min at 4 °C before plating in complete medium, defined as Dulbecco’s Modified Eagle Medium (DMEM) with 10% fetal calf serum (FCS) and 1% Penicillin/Streptomycin (P/S).

Induction of the inactive phenotype was performed by culturing HSCs in growth factor reduced (GFR) Matrigel (BD). Analysis was performed after 3 days in Matrigel for quantitative real time (qRT)-PCR. HSCs were cultured for 48 hours in media containing DMEM with 0.2% bovine serum albumin (BSA) and 1% P/S before treatment with TGF-β (2.5 ng/ml, R&D systems) for 16 hours. Doxepin and trimipramine (Microsource) were dissolved in DMSO. Amitriptyline and nortriptyline (Sigma) were dissolved in either DMSO or ethanol as indicated. B13 (Cayman) and C6-ceramide (Avanti) were dissolved in ethanol.

### Small molecule screen

On day 1 of the screen, 1500 cells were plated in 30 microliters of media per well of a 384-well plate. On day 3, 100 nanoliters of each compound were transferred by stainless steel pin array to each assay plate. On day 5, cells were fixed with 4% paraformaldehyde (PFA Electron Microscopy Sciences) followed by addition of Bodipy 493/503 (Life Technologies) and Hoechst 33342 (Thermo Scientific) stains. The cells were washed twice with dPBS and covered in 50 microliters of dPBS. The cells were imaged and analyzed using the Image Xpress Micro (IXM). Image analysis incorporated the total cell count (Hoechst positive) and total number of positive cells (Bodipy and Hoechst positive). The overall percentage of positive cells was calculated, as well as the mean, standard deviation, median, and Median Absolute Deviation (MAD) for each plate. Hits were determined by calculating (MAD) - based Z scores. The MAD was calculated by the median of the absolute difference between the percent positive cells and the median of the percent positive cells. The MAD-based z score was then calculated by (percent positive cells − median of percent positive cells)/(MAD*1.4826). If more than 60% cell death associated with a compound, the compound was excluded as a hit. We screened compounds from the NIH Clinical Collection 1-2013 and Biomol 4 libraries and half of the Microsource 1 library.

### Quantitative PCR Analysis

RNA was isolated from HSCs using Trizol Reagent (Life Technologies) and was reverse transcribed with iScript (Bio-Rad). Power SYBR Green master mix (Life Technologies) was used for quantification of cDNA on a CFX384 Real Time System (Bio-Rad). The following primers were used: forward *PPAR-γ*, 5′-TCAGTGGAGACCGCCCAG-3′, reverse *PPAR-γ*, 5′-TGGAGCTCCAGGGCTTGTAG-3′, forward *SMPD1*, 5′-ATGAAGCGATGGCCAAGGC-3′, reverse *SMPD1*, 5′-GAGACCGGGGTATGGGGAAA-3′, forward *ASAH1*, 5′ AGGGTTCCTCACTAGAACAGTTCT-3′, reverse *ASAH1*, 5′-ACAACCTTCCCCAGACTGGT-3′, forward *GAPDH*, 5′-ACAACTTTGGTATCGTGGAAGG-3′, reverse *GAPDH*, 5′-GCCATCACGCCACAGTTTC-3′, forward *COL1A1*, 5′-CAGGCTGGTGTGATGGGATT-3′, reverse *COL1A1*, 5′-AGCTCCAGCCTCTCCATCTT-3′, forward *ACTA2*, 5′-TCCCATCCATTGTGGGACGT-3′, reverse *ACTA2*, 5′-TTGCTCTGTGCTTCGTCACC-3′, forward *SPHK1*, 5′-AGGCTGAAATCTCCTTCACGC-3′, reverse *SPHK1*, 5′-GTCTCCAGACATGACCACCAG-3′, forward *SPHK2*, 5′-GGAGGAAGCTGTGAAGATGC-3′, reverse *SPHK2*, 5′-GCAACAGTGAGCAGTTGAGC-3′. *ACTA2, COL1A1, PPAR-γ, SMPD1, ASAH1, SPHK1* and *SPHK2* expression was normalized to *GAPDH*. All results are expressed as mean fold change ± SEM compared to controls.

### Microscopy

Cells were fixed with 4% PFA for 15 minutes and washed once with dPBS. Bodipy (67 pg/ul) and Hoescht (5 pg/ul) were diluted in dPBS and added to the wells. After 45 minutes, the cells were washed twice with dPBS, and imaged with a Nikon A1plus confocal microscope 40x lens. In each experiment, laser intensity, background level, contrast, and electronic zoom size were collected at the same level. Image processing was performed using Adobe Photoshop software.

### Western blot

HSCs were treated with 27 μM nortriptyline, 75 μM of B13, 25 μM of ceramide-C6, or ethanol vehicle for 48 hours and then washed in ice-cold dPBS and harvested in 100 ul of RIPA Buffer (Boston BioProducts) containing protease and phosphatase inhibitors (Sigma). Cellular lysates (30 mg) were prepared in Laemmli’s sample buffer (Boston BioProducts), separated by electrophoresis on a 12% polyacrylamide gel, and transferred to a polyvinylidene difluoride membrane (Millipore). Membranes were blocked with Tris-buffered saline 0.05% Tween 20 (TBS/T) containing 5% non-fat dry milk. Membranes were incubated with primary antibodies overnight at 4 °C and with secondary antibodies conjugated to horseradish peroxidase (HRP) (Cell Signaling Technology) for 1 hour at room temperature. Membranes were then washed three times with TBS/T. Immunoreactive bands were visualized on a C-Digit blot scanner (Li-Cor Biosciences) with a chemiluminescent HRP substrate (GE Healthcare). The following antibodies were used: αSMA (ab5694) and beta actin (ab8227) (Abcam).

### RNA interference

siGENOME small interfering RNAs (siRNA) were obtained from Dharmacon, and targeted human aSMase (GCAUAUAAUUGGCCACAUU, CUCUACAGGGCUCGAGAAA), aCDase (CACCAUAAAUCUUGACUUA, GAAAAUAGCACAAGUUAUG), sphingosine kinase 1 (GGGAAUUGAUGGUUAGCGA, CGACGAGGACUUUGUGCUA), and sphingosine kinase 2 (CAAGGCAGCUCUACACUCA, GCUCCUCCAUGGCGAGUUU). Non-targeting siRNA (siGENOME) from Dharmacon was used as a negative control (UAGCGACUAAACACAUCAA). Cells were plated in 48-well plates (20,000 cells/well) and were reverse transfected with 50 nM siRNA using DharmaFECT 1 (Dharmacon) in Dulbecco’s Modified Eagle Medium (DMEM) with 10% fetal calf serum (FCS) according to the manufacturer’s instructions. After 24 hours, siRNA was removed, and fresh medium was added. RNA was harvested for analysis after 48 hours.

### Preparation of RNA-seq libraries and expression analysis

RNA-seq analysis was performed on human HSC myofibroblasts treated with 27 μM nortriptyline and ethanol vehicle, or 25 μM ceramide-C6 and ethanol vehicle, for 48 hours. Analysis was also performed on human HSC myofibroblasts that underwent serum starvation for 48 hours followed by treatment with TGF-β (2.5 ng/ml) and either ethanol vehicle or 27 μM nortriptyline for 16 hours. Total RNA was isolated using Trizol reagent followed by DNAse I digestion and re-precipitation after phenol:chloroform and chloroform extractions. RNA quality was assessed via Agilent 2200 TapeStation, and samples with RNA integrity numbers (RINs) greater than 9 were used for library preparation. Isolated RNA was prepared for strand-specific sequencing according to TruSeq Stranded mRNA Library Prep Kit (Illumina). 600 ng of RNA was used for library construction. Two biological replicates were performed for each condition.

To identify the genes regulated by nortriptyline or ceramide treatment, we first mapped the RNA-seq for each condition to human reference genome (hg19) using Bowtie2 (v2.1.0)^64^ with *“-p4 –very-sensitive –phred33 –mm -D20 –score-min* = *C, -15,0 -q”* parameters. We then used HTSeq (with strand information) (v0.6.1)^65^ and DESeq2^66^ to identify the coding genes repressed or induced in nortriptyline-treated cells compared to vehicle-treated cells. Genes regulated by nortriptyline were defined by at least a 1.5-fold increase or decrease in the mean expression level in nortriptyline-treated cells compared to vehicle (FDR < 0.0001). Heatmaps were generated based on the RPKM values, which were calculated according to the HTSeq output. The same approach was adopted to identify the genes regulated by nortriptyline compared with vehicle in the presence of TGF-β.

### Gene Ontology (GO) Enrichment Analyses

GO enrichment analysis was performed using the protein-coding genes identified in differential expression analysis (http://david.abcc.ncifcrf.gov/)^67^,^68^. All protein-coding genes expressed with a minimum RPKM of 1.0 in HSCs were used as the background in the GO enrichment analyses.

### Co-expression network analysis

We used co-expression networks constructed from expression of protein-coding and HSC lncRNA genes (Spearman correlation > 0.7, p < 4e-7) across 47 human tissues and cell types, as previously described^26^. Protein-coding and lncRNA genes that are contained in the ECM cluster and decrease in expression in response to nortriptyline are shown. Cytoscape V3.1.1^69^ was used to visualize the connected networks and clusters.

### Sphingolipid Analysis

Human HSCs were treated with 27 μM nortriptyline or ethanol vehicle for 48 hours. Cells were then scraped and pelleted in cold PBS, and pellets were flash frozen. Lipids were extracted in 2 mL isopropanol:water:ethyl acetate (30:10:60 by volume).

Human HSCs were reverse transfected with a nontargeting siRNA or two independent siRNAs targeting aCDase as described above. Cellular lipids were extracted by modified Bligh and Dyer procedure^70^ with the use of 0.1 N HCl for phase separation. C17-S1P (30 pmols), C17-Sph (30 pmols), *N*-C17:0-Cer (30 pmols), *N*-12:0-sphingosylphosphorylcholine (12:0-SM, 100 pmols), *N*-D_3_-16:0-glucosylceramide (25 pmol) and *N*-D_3_-16:0-lactosylceramide (25 pmol) employed as internal standards, were added during the initial step of lipid extraction. The extracted lipids were dissolved in ethanol and aliquots were taken out to determine total phospholipid content as described by Vaskovsky *et al*.^71^. Samples were concentrated under stream of nitrogen, transferred into autosampler vials and subjected to consecutive LC-MS/MS analysis of sphingoid bases, ceramides, glycosyl/lactosyl ceramides, and sphingoid base-1-phosphates.

For all samples, cell extracts were analyzed by reverse phase high pressure liquid chromatography (HPLC) coupled to electrospray ionization and subsequent separation by mass spectrometry (MS). Analysis of sphingoid bases, ceramides and sphingomyelins was performed on a Thermo Quantum Ultra mass spectrometer, operating in a multiple reaction-monitoring positive ionization mode, as described^72^. Lipid phosphate concentrations were measured and sphingolipid levels were normalized to total lipid phosphate in each sample.

### *In vitro* Acid Sphingomyelinase Assay

Acid sphingomyelinase assays were performed as previously described^73^. In brief, 200 μM porcine brain sphingomyelin was mixed with [^14^C] labeled sphingomyelin and Triton X-100. Micelles were formed by sonication in a buffer containing 250 mM sodium acetate (pH 5.00) and 1.0 mM EDTA. Cells were lysed in buffer containing 50 mM Tris-HCl pH7.4, 0.2% Triton X-100, 1.0 mM EDTA, and protease inhibitor (SIGMAFAST™ Protease Inhibitor Cocktail Tablets, EDTA-Free, Sigma S8830). 25 μg of cell lysate in 100 μL lysis buffer was added to 100 μL of micelle mix and the reaction was incubated at 37 °C for 30 min. The reaction was terminated with the addition of 1.5 ml of CHCl3: MeOH (2:1, v/v) followed by 0.4 ml of water. Samples were vortexed, centrifuged (5 min at 3,000 rpm in a table top centrifuge), and 0.8 ml of the aqueous/methanolic phase was removed for scintillation counting.

### *In vitro* Acid Ceramidase Assay

To measure aCDase activity, cultured cells were collected and washed twice with PBS. Cell pellets were frozen in liquid nitrogen and stored at −80 °C for further use. Cell pellets were resuspended in 100 μl of 0.1 M sucrose solution and sonicated. Cell homogenates were centrifuged at 15,000 *g* for 3 min. The supernatant was collected and used for protein quantification to work with equal amounts of protein. The enzymatic assay was carried out in 96-well plates as previously described^74^. Briefly, for each well, 2 picomoles of RBM 14-12 substrate (Research Unit on Bioactive Molecule University of Barcelona) were resuspended in 50 μM of 2x reaction buffer (36 mM sodium acetate buffer pH4.5 substrate final concentration 20 μM) and combined with 50 μg of protein sample in a volume of 50 μl of the 0.1 M sucrose solution. The negative control consisted of the same incubation mixture in the absence of protein extracts. The plate was incubated at 37 °C for 3 h without agitation. The enzymatic reaction was stopped by adding 100 μl of a 2.5 mg/ml NaIO_4_ fresh solution in 100 mM glycine buffer pH 10.6 in each well. The released fluorescence was quantified using a microplate fluorescence reader (ex 360 nm, em 446 nm).

### Apoptosis Assay

Cells were analyzed for phosphatidylserine exposure using the Dead Cell Apoptosis Kit with Annexin V Alexa Fluor^®^ 488 & Propidium Iodide (ThermoFisher) according to manufacturer’s recommendations. A minimum of 25,000 cells were analyzed. Cell sorting was performed using a BD SORP 5 Laser FACS Vantage SE DIVA. Data were analyzed with FlowJo software.

### Statistics

All data are represented as the mean +/− standard error. All experiments were repeated a minimum of three times, unless otherwise stated. Statistical analysis was conducted using a two-tailed, paired Student’s *t* test or a two-way ANOVA test, as indicated. All statistical tests were performed with GraphPad Prism 6.0 (GraphPad Software Inc., La Jolla, CA, USA). Statistical significance was defined as P < 0.05.

### Data Availability

RNA-seq data produced for this study are available at GSE78853.

## Additional Information

**How to cite this article:** Chen, J. Y. *et al*. Tricyclic Antidepressants Promote Ceramide Accumulation to Regulate Collagen Production in Human Hepatic Stellate Cells. *Sci. Rep.* **7**, 44867; doi: 10.1038/srep44867 (2017).

**Publisher's note:** Springer Nature remains neutral with regard to jurisdictional claims in published maps and institutional affiliations.

## Supplementary Material

Supplementary Materials

Supplementary Dataset 1

Supplementary Dataset 2

Supplementary Dataset 3

## Figures and Tables

**Table 1:** Results of a small molecule screen to identify compounds that inhibit hepatic fibrosis.

Small Molecule	Average MAD- based Z score	Activity
Merbromin	58.8	Antiseptic
Vinblastine	17.8	Binds tubulin
Pararosaniline	17.5	Magenta solid
Nortriptyline^†^	12.7	TCA
Dibucaine	11.3	Anesthetic
Doxepin^†^	9.6	TCA
Fluoxetine	9.2	SSRI
Trimeprazine	8.8	Antihistamine
Clemastine	8.0	Antihistamine
Colchicine	7.8	Binds tubulin
Mesoridazine	7.8	Antipsychotic
Guanabenz	7.7	a2 R agonist
Paroxetine	7.5	SSRI
Emetine	7.0	Antiprotozoal
Trimipramine^†^	6.4	TCA
Mefloquine	6.3	Antimalarial
Chlorprothixene	6.3	Antipsychotic
Proadifen	6.1	Inhibits P450
Cortisone	5.9	Glucocorticoid
Imipramine^†^	5.7	TCA
Chloroquine	5.4	Antiprotozoal

A list of the 21 hits identified by the screen ranked by average MAD-based Z score. TCAs: Tricyclic antidepressants; SSRI: selective serotonin reuptake inhibitors; a2 R agonist: alpha 2 adrenergic receptor agonist. ^†^Denotes TCAs.
