# Supplementary material for: Tricyclic Antidepressants Promote Ceramide Accumulation to Regulate Collagen Production in Human Hepatic Stellate Cells
Source: Sci Rep. 2017 Mar 21;7:44867. doi: 10.1038/srep44867 (PMC5359599; doi:10.1038/srep44867)
Supplement: Supplementary Materials [file srep44867-s1.pdf]

Tricyclic Antidepressants Promote Ceramide Accumulation to Regulate Collagen Production in Human Hepatic Stellate Cells

Authors: Jennifer Y. Chen, Benjamin Newcomb, Chan Zhou, Joshua V. Pondick, Sarani Ghoshal, Samuel York, Daniel Motola, Nicolas Coant, Jae Kyo Yi, Cungui Mao, Kenneth K. Tanabe, Irina Bronova, Evgeny V. Berdyshev, Bryan C. Fuchs, Yusuf Hannun, Raymond T. Chung*, Alan C. Mullen*

*These authors contributed equally to this work.

Supplementary Materials

Supplemental Fig. 1. TCA treatment inhibits expression of protein-coding and long noncoding RNAs linked to fibrosis.

Supplemental Fig. 2. Depletion of aCDase results in decreased aCDase activity.

Supplemental Fig. 3. ACDase inhibition and addition of ceramide modulate genes that regulate fibrosis in human HSCs and do not result in significant apoptosis.

Data files S1. Gene expression regulated by nortriptyline and ceramide.

Data file S2. Sphingolipid analysis of HSCs treated with ethanol vehicle or nortriptyline for 48 hours.

Data file S3. Sphingolipid analysis of HSCs treated with siRNAs targeting acid ceramidase or a nontargeting siRNA.

Supplemental Figure 1

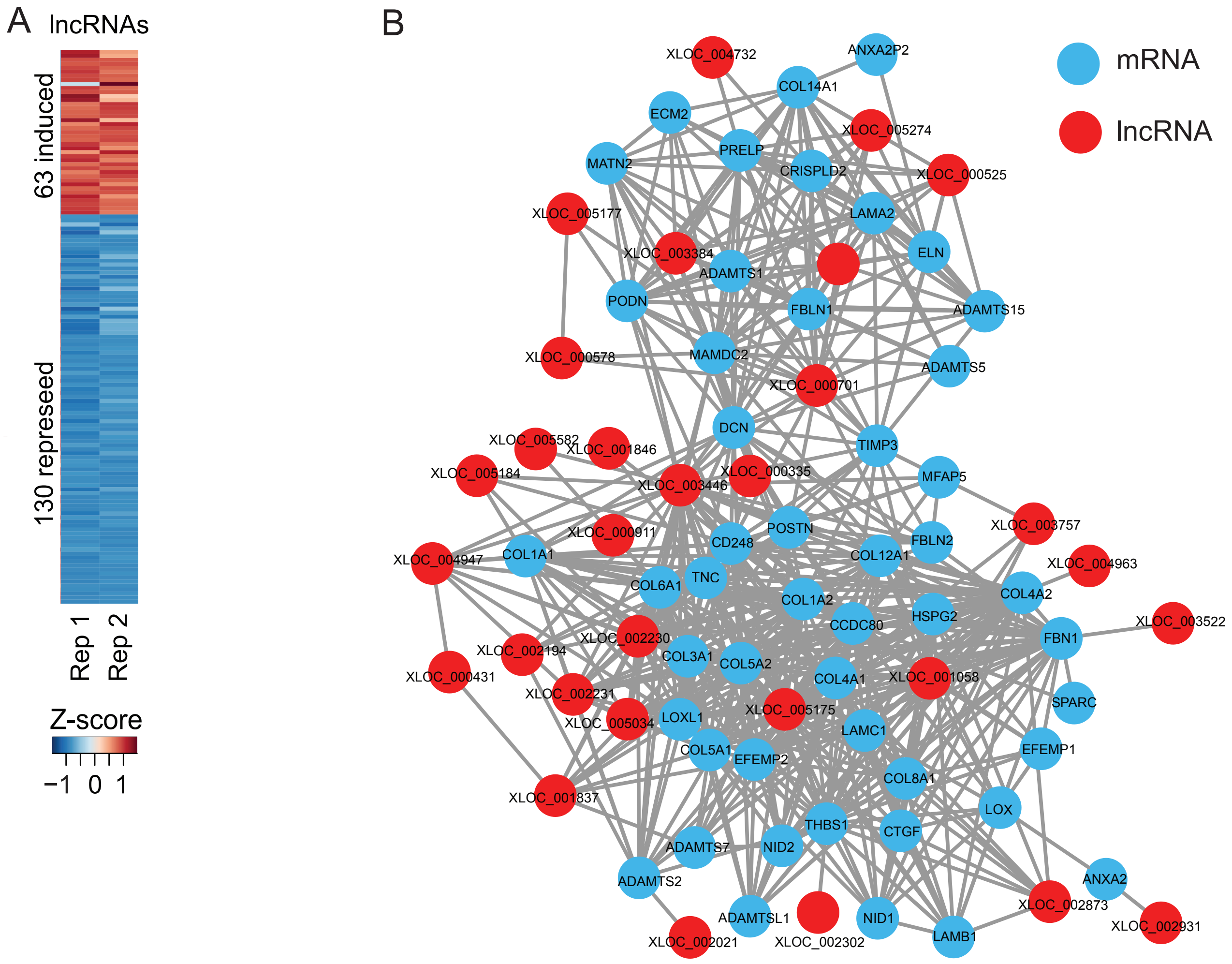

Supplemental Fig. 1. TCA treatment inhibits expression of protein-coding and long noncoding RNAs linked to fibrosis. (A) lncRNAs expressed in HSCs that were induced (red) or repressed (blue) after nortriptyline treatment compared to vehicle are shown (at least 1.5 fold change in expression, false discovery rate <0.0001). Relative expression (Z-score) for two replicates (Rep 1 and Rep 2) are shown. **(B)** 50 ECM genes and 31 lncRNAs that are repressed by nortriptyline are contained in the ECM network (spearman correlation >0.7 and p<4e-7[26]). Each gray line in the network connects two genes that are co-expressed.

Supplemental Figure 2

A

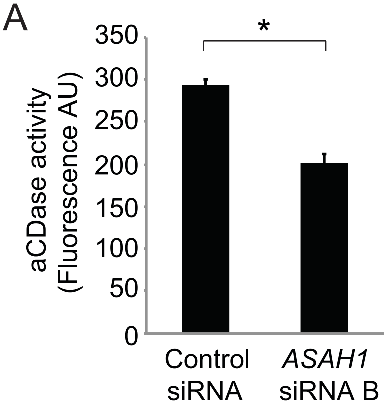

Supplemental Fig. 2. Depletion of aCDase results in decreased aCDase activity. ACDase enzyme assay results of HSCs transfected with nontargeting siRNA (control) or siRNA targeting aCDase mRNA (*ASAH1*). *$p < 0.05$

Supplemental Figure 3

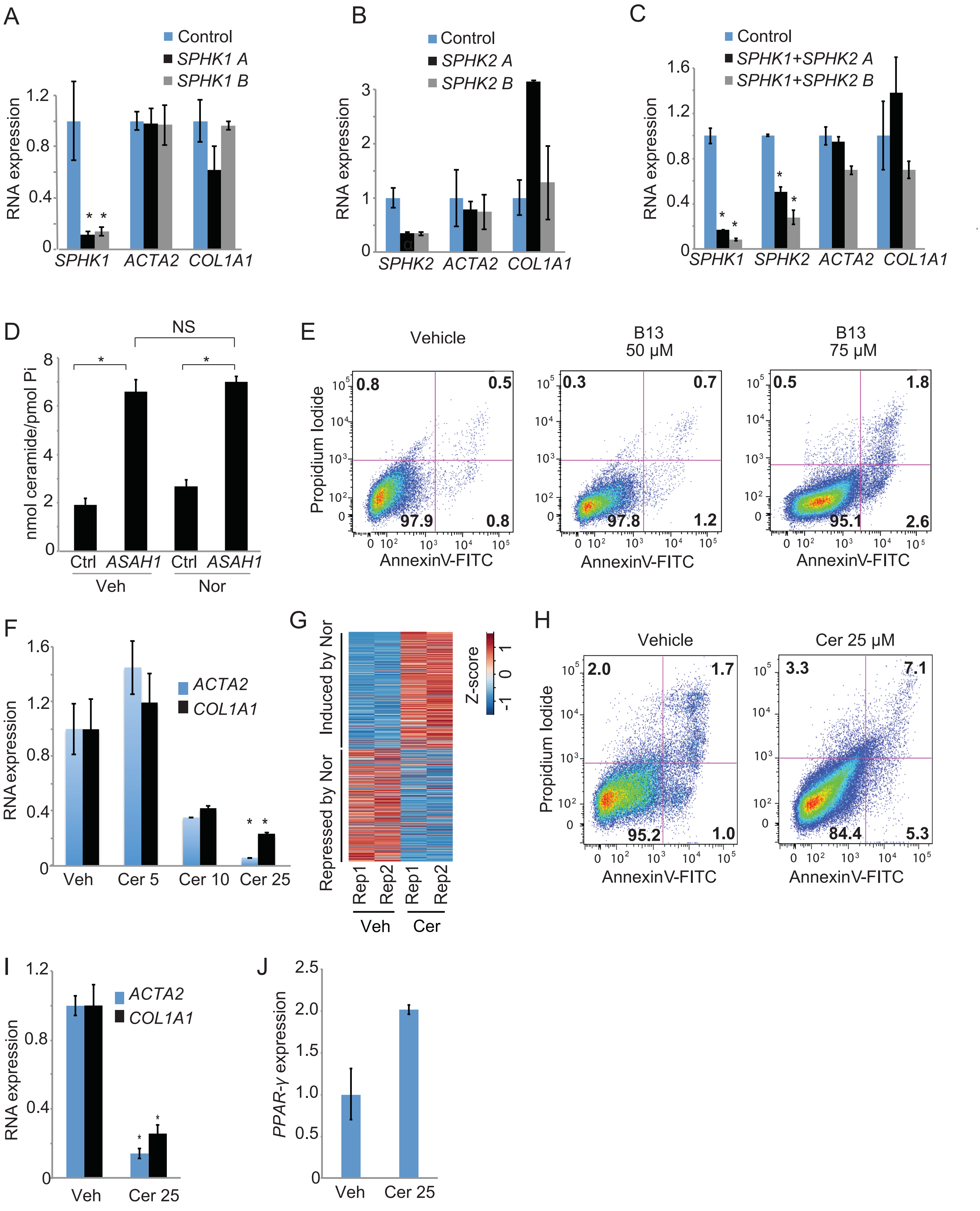

Supplemental Fig. 3. ACDase inhibition and addition of ceramide modulate genes that regulate fibrosis in human HSCs and do not result in significant apoptosis. (A, B and C) qRT-PCR analysis was performed to quantify expression of the indicated genes after transfection with nontargeting siRNA (control) and siRNAs targeting sphingosine kinase 1 (*SPHK1*) and sphingosine kinase 2 (*SPHK2*) separately (A and B) or in combination (C). Two different siRNAs (A and B) are used for each mRNA. Samples were normalized using *GAPDH*. * $p < 0.05$ **(D)** Ceramide was measured in HSCs transfected with a nontargeting siRNA (control) or siRNAs targeting aCDase mRNA (*ASAH1*) for 48 hours followed by the addition of ethanol vehicle (Veh) or nortriptyline (Nor, 27 μM) for 24 hours. * $p < 0.05$, NS = not significant ($p \geq 0.05$) **(E)** Flow cytometry was performed using HSCs after treatment with ethanol vehicle, 50 μM of B13, and 75 μM of B13. The percentages of apoptotic (AnnexinV+ Propidium Iodide−) and necrotic (AnnexinV+ Propidium Iodide+) are indicated within the quadrants. **(F)** qRT-PCR analysis was performed to quantify expression of the indicated genes after addition of ceramide-C6 at the indicated concentrations. Samples were normalized using *GAPDH*. * $p < 0.05$ **(G)** Changes in gene expression are shown in response to ceramide treatment. The protein-coding genes induced and repressed by nortriptyline are listed in the same order as in Fig. 3A. The protein-coding genes induced and repressed by nortriptyline are listed in the same order as in Fig. 3A. The heatmaps show the change in expression between ceramide and vehicle treatment for genes regulated by nortriptyline. Red indicates increased expression with ceramide treatment and blue indicates decreased expression. The relative expression (Z-score) of two replicates (Rep 1 and Rep 2) is shown. **(H)** Flow cytometry was performed using HSCs after treatment with ethanol vehicle and 25 μM of ceramide-C6. The percentages of apoptotic (AnnexinV+ Propidium Iodide−) and necrotic (AnnexinV+ Propidium Iodide+) are indicated within the quadrants. **(I, J)**

qRT-PCR analysis was performed to quantify expression of the indicated genes after addition of ethanol vehicle (Veh) or Ceramide-C6 (25 μM) followed by sorting of cells that were negative for Annexin V and Propidium Iodide staining. Samples were normalized using *GAPDH*. * $p < 0.05$. $p=0.05$ for change in *PPAR-γ* expression.
